# Fructose‐1,6‐bisphosphate aldolase of *Neisseria meningitidis* binds human plasminogen via its C‐terminal lysine residue

**DOI:** 10.1002/mbo3.331

**Published:** 2016-01-05

**Authors:** Fariza Shams, Neil J. Oldfield, Si Kei Lai, Sarfraz A. Tunio, Karl G. Wooldridge, David P. J. Turner

**Affiliations:** ^1^ School of Life SciencesMolecular Bacteriology and Immunology GroupCentre for Biomolecular SciencesUniversity of NottinghamNottinghamNG7 2RDUnited Kingdom; ^2^ Present address: Department of MicrobiologyUniversity of SindhPakistan

**Keywords:** Aldolase, *Neisseria meningitidis*, pathogenesis, plasminogen, protein moonlighting.

## Abstract

*Neisseria meningitidis* is a leading cause of fatal sepsis and meningitis worldwide. As for commensal species of human neisseriae, *N. meningitidis* inhabits the human nasopharynx and asymptomatic colonization is ubiquitous. Only rarely does the organism invade and survive in the bloodstream leading to disease. Moonlighting proteins perform two or more autonomous, often dissimilar, functions using a single polypeptide chain. They have been increasingly reported on the surface of both prokaryotic and eukaryotic organisms and shown to interact with a variety of host ligands. In some organisms moonlighting proteins perform virulence‐related functions, and they may play a role in the pathogenesis of *N. meningitidis*. Fructose‐1,6‐bisphosphate aldolase (FBA) was previously shown to be surface‐exposed in meningococci and involved in adhesion to host cells. In this study, FBA was shown to be present on the surface of both pathogenic and commensal neisseriae, and surface localization and anchoring was demonstrated to be independent of aldolase activity. Importantly, meningococcal FBA was found to bind to human glu‐plasminogen in a dose‐dependent manner. Site‐directed mutagenesis demonstrated that the C‐terminal lysine residue of FBA was required for this interaction, whereas subterminal lysine residues were not involved.

## Introduction

*Neisseria meningitidis* is a prominent cause of bacterial meningitis and severe sepsis. The organism asymptomatically colonizes the nasopharyngeal mucosa, especially in young adults. In susceptible individuals, hyper‐invasive strains of meningococci may invade the nasopharyngeal submucosa and subsequently enter the bloodstream (Stephens 2009). Diverse bacterial factors, involved in adhesion, invasion, dissemination, and protection of the organism from the innate human immune system, are expressed by *N. meningitidis*. These include the polysaccharide capsule, lipooligosaccharide, type IV pili, and a large number of outer membrane or secreted proteins such as Opa, Opc, fHbp, NspA, App, MspA, NalP, HrpA‐HrpB, NhhA, NHBA, NadA, and the porin proteins, PorA and PorB (Virji 2009; Hill and Virji 2012; Pizza and Rappuoli 2015).

Moonlighting proteins are increasing recognized for their roles in bacterial pathogenesis (Henderson and Martin 2011). By definition, moonlighting proteins perform two or more biochemical functions using a single polypeptide chain (Jeffery 1999). Commonly they are enzymatically active with cytoplasmic roles in glycolysis or other metabolic pathways, or have roles in protein synthesis (elongation factor Tu) or protein stabilization and folding (DnaK), but are also displayed on the bacterial surface where they perform functions unrelated to their cytoplasmic roles, often by interacting with specific host molecules (Wang et al. 2013). In meningococci, several putative moonlighting proteins have been described including enolase, peroxiredoxin, and DnaK (Knaust et al. 2007), GapA1 (Tunio et al. 2010a), and fructose‐1,6‐bisphosphate aldolase (FBA) (Tunio et al. 2010b). Plasminogen binding on the meningococcal cell surface was first described by Ullberg and colleagues (Ullberg et al. 1992), and is a widely recognized phenomenon in bacterial pathogens (Lähteenmäki et al. 2005; Sanderson‐Smith et al. 2012). In meningococci, enolase, peroxiredoxin, and DnaK were previously shown to bind plasminogen on the bacterial surface (Knaust et al. 2007).

Plasminogen (plg) is a 92‐kDa plasma glycoprotein proenzyme; posttranslational processing results in several different forms, but the circulating mature form is known as glu‐plasminogen (glu‐plg) reflecting the presence of an N‐terminal glutamic acid residue. Glu‐plg is composed of a small N‐terminal preactivation peptide, five homologous disulfide‐bonded triple‐loop kringle domains (K1‐5) and a serine protease (SP) domain (Law et al. 2012). Kringle domains 1–4 occur naturally as a 38‐kDa fragment of plasminogen, which is known as angiostatin due to its inhibitory effect on angiogenesis (Abad et al. 2002). The last kringle domain (K5), together with the SP domain, is known as mini‐plasminogen. Kringle domains contain multiple lysine‐binding sites (LBS); intramolecular binding between lysine residues and the LBS maintains glu‐plg in a closed, activation‐resistant, conformation (Ponting et al. 1992). Competitive binding with interaction partners such as fibrin and cell surface receptors, allows glu‐plg to adopt an open conformation, exposing an activation loop to cleavage by activators, such as tissue‐type plasminogen activator (tPA) or urokinase‐type plasminogen activator (uPA), to form plasmin (Lijnen 2001). Plasmin is a broad‐spectrum serine protease which plays an essential role in hemostasis by degrading fibrin clots (Lijnen 2001). In addition, plasmin also cleaves extracellular matrix proteins including fibronectin, vitronectin, and laminin (Plow et al. 1995), and activates collagenases, matrix metalloproteinases and latent macrophage elastase, resulting in the cleavage of elastin, collagen, and proteoglycans (Murphy et al. 1999). Plasmin was also recently shown to be a potent inhibitor of the complement components C3 and C5 (Barthel et al. 2012a).

Bacteria can interfere with and manipulate the plg system by two general mechanisms: firstly by producing activators of plg (e.g., streptokinase produced by *Streptococcus pyogenes*) (McArthur et al. 2012) and secondly by immobilizing plg (either directly or indirectly) on the bacterial cell surface, which may then be activated to plasmin by activators (Sanderson‐Smith et al. 2012). Examples of bacterial surface‐localized plg receptors include streptococcal *α*‐enolase (Pancholi and Fischetti 1998), *Escherichia coli* fimbriae (Kukkonen et al. 1998), *Haemophilus influenzae* aspartase (Sjöström et al. 1997), and protein E (Barthel et al. 2012b). Sequestered plg contributes to processes such as ECM degradation, fibrinolysis, degradation of immune effectors and adherence, thus enhancing bacterial colonization of, and dissemination within, the host (Bhattacharya et al. 2012).

We previously reported that FBA is a nonessential, surface‐localized protein in *N. meningitidis*, which belongs to the Class II subfamily of FBAs (Tunio et al. 2010b). Importantly, FBA is required for optimal association of meningococcal cells to human epithelial and endothelial cells (Tunio et al. 2010b). More recently, FBA of *Mycobacterium tuberculosis* was shown to be an essential enzyme and partly localized to the bacterial surface where it contributes to plg binding (de la Paz Santangelo et al. 2011). In this report, we further examine the properties and role of FBA on the cell surface of neisseriae. We demonstrate that: FBA is present on the surface of pathogenic and nonpathogenic species of neisseriae; aldolase activity is not required for cell surface localization or anchoring of FBA; and that FBA binds human plg, principally via the C‐terminal lysine residue.

## Experimental Procedures

### Bacterial strains

*Escherichia coli* JM109 (Table S1) was used for the expression of 6 × histidine‐tagged rFBA and derivatives. *E. coli* XL10‐Gold ultracompetent cells were used as a host strain for the construction of mutagenic plasmids. *E. coli* strains were grown at 37°C in Lysogeny Broth (LB) broth or on LB agar supplemented, where appropriate, with ampicillin (100 *μ*g mL^−1^), kanamycin (30 *μ*g mL^−1^) or erythromycin (200 *μ*g mL^−1^). Strains of Neisseria (Table S1) were grown at 37°C in air plus 5% CO_2_ on chocolated horse blood agar (Oxoid), Brain Heart Infusion (BHI) agar or BHI broth supplemented with 1% Vitox (Oxoid) and kanamycin (50 *μ*g mL^−1^) or erythromycin (5 *μ*g mL^−1^) where appropriate.

### DNA manipulation

Genomic DNA was extracted from *N. meningitidis* using the DNeasy Tissue kit (Qiagen, Manchester, UK). Plasmid DNA was prepared by using the QIAprep Spin kit (Qiagen). DNA was quantified using a NanoDrop 1000 Spectrophotometer (NanoDrop Technologies, Wilmington, Delaware, USA). Restriction enzymes were purchased from New England Biolabs. All enzymatic reactions were carried out according to the manufacturer's instructions. A Rapid DNA Ligation kit (Fermentas Life Sciences, Vilnius, Lithuania) was used for ligation reactions. DNA sequencing was carried out by Source Bioscience, UK.

### *Construction of meningococcal* cbbA *mutants*

*N. meningitidis cbbA* mutants were obtained by natural transformation and allelic exchange utilizing a previously described mutagenesis plasmid (pSAT‐4; Table S2) (Tunio et al. 2010b). Replacement of *cbbA* with a kanamycin resistance cassette in mutant strains was confirmed by PCR and the absence of FBA expression confirmed by immunoblot analysis.

### SDS‐PAGE and immunoblotting

Proteins were electrophoretically separated using 10% polyacrylamide gels (Mini‐Protean III; Bio‐Rad, Hemel Hempstead, UK) and were stained using SimplyBlue Safestain (Invitrogen, Waltham, Massachusetts, USA) or transferred to nitrocellulose membranes (Schleicher & Schuell) by using a Trans‐Blot SD semidry transfer cell (Bio‐Rad) according to the manufacturer's recommendations. Membranes were probed with mouse antipentahistidine antibody (Qiagen) or rabbit anti‐FBA primary antibody (*α*R‐FBA) diluted 1:10,000 or 1:1000, respectively, in blocking buffer [5% (wt/vol) nonfat dry milk, 0.1% (vol/vol) Tween 20 in 1 × phosphate‐buffered saline (PBS)] and incubated for 2 h. After washing in PBS with 0.1% Tween 20 (PBST), membranes were incubated for 2 h with 1:10,000‐diluted goat anti‐mouse (or anti‐rabbit) IgG‐alkaline phosphatase conjugate (Sigma‐Aldrich, Gillingham, Dorset, UK). After washing with PBST, blots were developed using BCIP/NBT‐Blue liquid substrate (Sigma‐Aldrich).

### Flow cytometry

Samples were prepared for flow cytometry as described previously (Tunio et al. 2010b). Briefly, *N. meningitidis* strains were grown to OD_600_ ~0.7, and 1 × 10^7^ cfu aliquots were centrifuged at 5000*g* for 5 min and resuspended in 0.2 mL filtered PBS. Cells were incubated for 2 h with *α*R‐FBA (1:500 diluted in PBS containing 0.1% BSA, 0.1% sodium azide and 2% fetal calf serum) and untreated cells were used as a control. Cells were washed with PBS and incubated for 2 h in the dark with goat anti‐rabbit IgG‐Alexa Fluor488 conjugate (Invitrogen; diluted 1:50). Again, untreated cells were used as a control. Finally, the samples were washed in PBS before being resuspended in 1 mL of PBS containing 0.5% formaldehyde. Samples were analyzed for fluorescence using a BD FACSAria flow cytometer and BD FACS Diva software (BD Biosciences, Oxford, UK). Cells were detected using forward and side scatter dot plots and bacterial aggregates and cell debris were excluded by defining a gating region. A total of 20,000 events were analyzed for fluorescence signals. Subsequent data analysis was done using WinMDI 2.9 software (Mybiosoftware, Provo, Utah, USA). For determination of plasminogen binding by neisserial cells, the cells were incubated with glu‐plasminogen (3 *μ*g mL^−1^; Merck Millipore, Damstadt, Germany) for 1 h at 37°C. Cells were washed three times with PBS and incubated with goat anti‐plasminogen antibody (1:100). After washing, donkey anti‐goat IgG‐Alexa Fluor488 conjugate (1:50 diluted) was added, and after further washing, cells were fixed and analyzed as previously described.

### Site‐directed mutagenesis of FBA residue D83

A single‐nucleotide mutation (A–C) at position 248 of *cbbA* was introduced into pSAT‐9 (Table S2; for expression of rFBA^D83A^ in *E. coli*) and pSAT‐12 (Table S2; for the expression of FBA^D83A^ in *N. meningitidis*) using the QuikChange Lightning site‐directed mutagenesis kit (Agilent Technologies Santa Clara, California, USA). Reactions were undertaken following the manufacturer's instructions and utilizing primers FBA_D83AF and FBA_D83AR (Table S3). The plasmid resulting from the mutagenesis of pSAT‐12 (pFS‐5; Table S2), was used to transform MC58Δ*cbbA* by natural transformation, thus introducing a single chromosomal copy of the mutated *cbbA* allele (encoding FBA^D83A^) and the downstream erythromycin resistance cassette in the intergenic region between NMB0102 and NMB0103, generating MC58Δ*cbbA cbbA* ^*EctD83A*^. Insertion of the mutated *cbbA* gene and erythromycin resistance cassette at the ectopic site was confirmed by PCR analysis and sequencing. Immunoblot analysis confirmed expression of FBA in MC58Δ*cbbA cbbA* ^*EctD83A*^ at similar levels to wild‐type MC58 or MC58Δ*cbbA* complemented with a wild‐type copy of *cbbA* (MC58Δ*cbbA cbbA* ^*Ect*^).

### Expression and purification of recombinant FBA

All rFBA derivatives were expressed and purified as previously described (Tunio et al. 2010b). Briefly, *E. coli* cell pellets were resuspended in 20 mL lysis buffer (50 mmol L^−1^ NaH_2_PO_4_, 300 mmol L^−1^ NaCl, 10 mmol L^−1^ imidazole; pH 7.4) followed by a 10 min cycle of 30 sec sonication and 30 sec off, on ice. The cell lysate was centrifuged (4000*g* for 10 min) and the cleared lysate was loaded onto a HisTrap FF column (GE Healthcare Lifesciences) prepacked with Ni Sepharose six Fast Flow (GE Healthcare Lifesciences, Little Chalfont, Buckinghamshire, UK) connected to a ÄKTAprime plus liquid chromatography system (GE Healthcare Lifesciences), equilibrated with 10 column volumes of wash buffer (50 mmol L^−1^ NaH_2_PO_4_, 300 mmol L^−1^ NaCl, 15 mmol L^−1^ imidazole; pH 7.4). Proteins were eluted by step elution using elution buffer (50 mmol L^−1^ NaH_2_PO_4_, 300 mmol L^−1^ NaCl, 300 mmol L^−1^ or 500 mmol L^−1^ imidazole; pH 7.4). HiTrap column prepacked with 5 mL of Sephadex G‐25 Superfine (GE Healthcare Lifesciences) equilibrated with 5 column volumes of phosphate‐buffered saline (PBS) was used for buffer exchange. Eluted fractions were collected in 50% glycerol. Chromatography was analyzed using Prime view 5.0 software (Amersham Biosciences, Little Chalfont, Buckinghamshire, UK). Purified elutes were concentrated using Vivaspin sample concentrators (Sartorius, Epsom, Surrey, UK; 10,000 MWCO). The kinetic analysis of fructose bisphosphate aldolase activity of rFBA and rFBA^D83A^ was done using a previously described methodology (Tunio et al. 2010b).

### Association assays

Association assays were performed essentially as previously described (Oldfield et al. 2007). Briefly, human brain microvascular endothelial (HBME) cells were grown to confluence in Dulbecco's Modified Eagle's Medium (DMEM) supplemented with 10% heat‐inactivated fetal calf serum (FCS; Invitrogen) and 2% antibiotic antimycotic solution (Invitrogen) in 24‐well tissue culture plates (Costar, Sigma‐Aldrich) at 37°C in an atmosphere of 5% CO_2_. Prior to all experiments, monolayers were transferred to DMEM supplemented with 2% FCS. Meningococci were cultured for 2 h, and monolayers were infected with 1 × 10^7^ cfu of meningococci and cultured for 2 h in 5% CO_2_ at 37°C. Monolayers were washed four times with PBS and then disrupted and homogenized in 1 mL 0.1% saponin in PBS. Meningococci were enumerated by serial dilution of the homogenized suspensions and subsequent determination of colony‐forming units by plating 50 *μ*L aliquots from appropriate dilutions of the lysates on agar. All assays were repeated at least three times. Statistical significance was measured using the two‐tailed Student's *t*‐test. *P* value of ≤0.05 was considered significant.

### ELISA

Microplate wells (Nunc 96‐well plates, PolySorp) were coated with 100 *μ*L volumes of 0.5 or 5 *μ*g mL^−1^ glu‐plg (from human plasma; Calbiochem), laminin (human placenta; Merck Millipore), fibronectin (human plasma; Sigma) or collagen I (human placenta; Corning) in 0.2 *μ*m‐filtered sodium carbonate buffer (142 mmol L^−1^ NaHCO_3_, 8 mmol L^−1^ Na_2_SO_3_, pH 9.0) and incubated for 1 h. After washing five times with PBS‐Tween (0.05%; PBST), wells were blocked with 100 *μ*L of 0.2 *μ*m‐filtered 1% BSA in PBS for 1 h. After removal of the blocking solution, 100 *μ*L of 5 *μ*g mL^−1^ rFBA in 1% BSA/PBS was added and incubated for 1 h. Following vigorous washing with PBST, 100 *μ*L of mouse antipentahistidine antibody (Qiagen; diluted 1:2000 in 1% BSA/PBS) was added for incubation for 1 h. Plates were again vigorously washed, before the addition of 100 *μ*L goat anti‐mouse IgG‐horse radish peroxidase conjugate (Sigma‐Aldrich; diluted 1:10,000 in 1% BSA/PBS), was added and incubated for 1 h. Plates were again vigorously washed, and the color was developed by adding 100 *μ*L ABTS substrate (Roche Applied Science, Burgess Hill, West Sussex, UK), and absorbance at 405 nm was measured using a Biotek EL800 spectrophotometer. Values shown are minus the values obtained from control wells coated with 1% BSA.

Alternatively, binding assays were undertaken as above, but using rFBA or its derivatives (10 *μ*g mL^−1^) as the immobilized ligand to capture glu‐plg (10 *μ*g mL^−1^). Bound glu‐plg was detected using goat anti‐human plg (1:20,000; Rockland Immunochemicals, Pottstown, Pennsylvannia, USA) and donkey anti‐goat IgG‐alkaline phosphatase conjugate (1:5,000; Promega Southampton, Hampshire, UK). After washing, 200 *μ*L of phosphatase substrate (Sigma), dissolved in buffer containing 0.1 mol L^−1^ glycine, 1 mmol L^−1^ ZnCl_2_ and 1 mmol L^−1^ MgCl_2_, pH 10.4, was added into each well and readings taken. The lysine analog, ε‐aminocaproic acid (EACA; 5 mmol L^−1^) (Sigma‐Aldrich) was utilized for inhibition studies.

### Cloning of truncated rFBA (tr‐rFBA) and mutagenesis of C‐terminal lysine residues

A 372 bp fragment of *cbbA* encoding the C‐terminal 123 amino acids was amplified from MC58 meningococcal DNA using the primers CterFBAF1 and CterFBAR1 (Table S3). The amplified fragment and pQE‐30 (Table S2) were digested using BamHI according to the manufacturer's instructions and ligated to form pFS‐7 encoding N‐terminally histidine tagged tr‐rFBA. The FBA lysine residues (^339^K, ^346^K, and ^354^K) were replaced by alanine residues using the QuikChange Lightning site‐directed mutagenesis kit (Agilent Technologies) following the manufacturer's instructions, yielding pFS‐71, pFS‐72, and pFS‐73, respectively (Table S2). Primers utilized are given in Table S3.

## Results

### FBA is present on the surface of *N. gonorrhoeae* and commensal neisseriae

**Figure 1:**
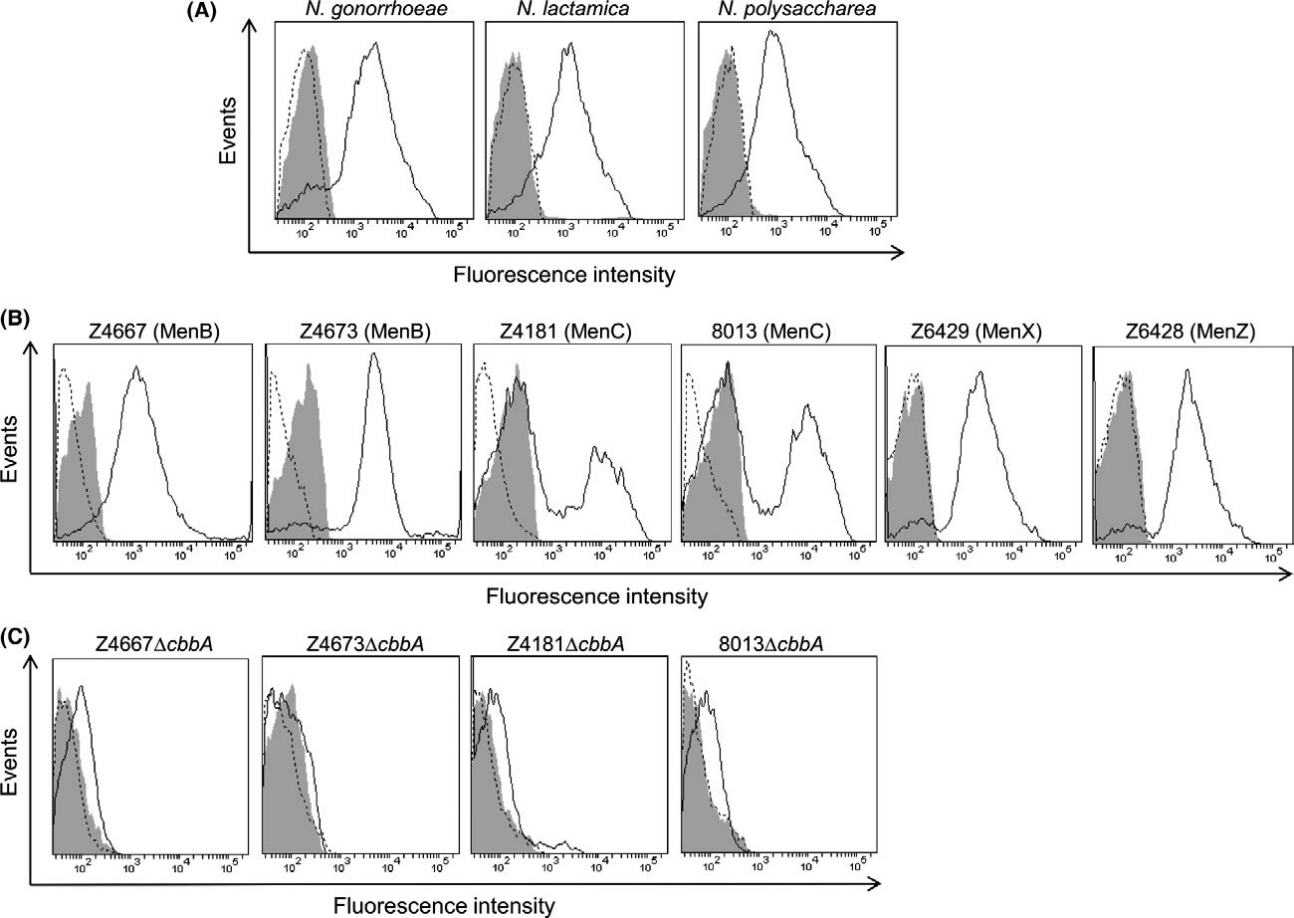
Flow cytometric analysis of Fructose‐1,6‐bisphosphate aldolase (FBA) surface localization in neisseriae. Presence of FBA on the cell surface was detected by anti‐FBA antiserum and anti‐rabbit IgG‐Alexa Fluor488. Fluorescence is displayed as a histogram: area enclosed by the dashed line indicates bacteria treated with primary antibody only; area shaded in gray indicates bacteria treated with secondary only; area enclosed by the solid line indicates bacteria treated with both antibodies. (A). FBA detected on the surface of *N. gonorrhoeae, N. lactamica,* and *N. polysaccharea*. (B). The presence of FBA confirmed on the surface of six wild‐type *N. meningitidis* strains (serogroups in parentheses). (C). Detection of surface‐localized FBA was abolished in *N. meningitidis cbbA* mutant derivatives.

We previously showed that FBA is expressed by a diverse collection of meningococcal isolates and by strains of *N. gonorrhoeae*,* N. lactamica,* and *N. polysaccharea*; flow cytometry confirmed the presence of FBA on the surface of the meningococcal serogroup B (MenB) strain MC58 (Tunio et al. 2010b). Here, *N. gonorrhoeae* FA1090, *N. lactamica* ATCC 23970, and a clinical isolate of *N. polysaccharea* were subjected to flow cytometry analysis to detect surface‐localized FBA. All three strains, when treated with both primary and secondary antibody, demonstrated a clear shift in fluorescence signal indicating FBA surface localization (Fig. 1A). We also examined FBA surface localization in six additional meningococcal strains: two MenB; two serogroup C (MenC); one serogroup X (MenX); and one serogroup Z (MenZ). Surface exposure of FBA was demonstrated in all strains examined (Fig. 1B). No shift in fluorescence signal was observed following inactivation of the gene encoding FBA (*cbbA*) in the MenB and MenC isolates, confirming antibody specificity (Fig. 1C). Interestingly, reduced levels of fluorescently labeled bacteria were observed for the wild‐type MenC strains (41.9% for Z4181; 48% for 8013, respectively) compared to the other meningococcal strains (Fig. 1B; all ≥77%). Taken together, our data suggest that FBA is commonly present on the cell surface of meningococcal strains and also in other neisseriae species, including commensals.

### FBA surface localization and moonlighting functions are not dependent on aldolase activity

**Figure 2:**
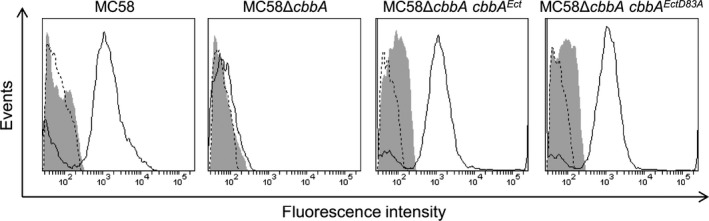
Aldolase activity is not required for surface localization or anchoring of meningococcal Fructose‐1,6‐bisphosphate aldolase (FBA). FBA lacking aldolase activity (FBA^D83A^) could be detected on the cell surface of MC58Δ*cbbA cbbA* ^*EctD83A*^ cells. Fluorescence is displayed as a histogram: area enclosed by the dashed line indicates bacteria treated with primary antibody only; area shaded in gray indicates bacteria treated with secondary only; area enclosed by the solid line indicates bacteria treated with both antibodies.

Mutation of a specific aspartate residue (^109^D) in the cofactor‐binding site of *E. coli* class II FBA abolishes aldolase activity (Plater et al. 1999). We replaced the corresponding residue (^83^D) in recombinant meningococcal FBA (rFBA) with alanine. A coupled enzymic assay, which we previously used to estimate the kinetic parameters of rFBA for cleavage of fructose bisphosphate (FBP) (Tunio et al. 2010b), was used to confirm that purified rFBA^D83A^ exhibited no detectable aldolase activity (data not shown). We next introduced a mutated *cbbA* allele encoding FBA^D83A^ into *N. meningitidis* to investigate the effect of abolishing aldolase activity on FBA surface localization and moonlighting functions. To achieve this, site‐directed mutagenesis was used to alter *cbbA* harbored by pSAT‐12, previously used to insert a wild‐type copy of *cbbA* into MC58Δ*cbbA* at an ectopic site (Tunio et al. 2010b), to create pFS‐5. Natural transformation and allelic exchange of MC58Δ*cbbA* utilizing pFS‐5 yielded MC58Δ*cbbA cbbA* ^*EctD83A*^. *N. meningitidis* MC58 wild‐type, MC58Δ*cbbA* and MC58Δ*cbbA* complemented with either wild‐type (MC58Δ*cbbA cbbA* ^*Ect*^) or mutated (MC58Δ*cbbA cbbA* ^*EctD83A*^) *cbbA* alleles were then subjected to flow cytometry analysis. As expected, wild‐type MC58 and MC58Δ*cbbA cbbA* ^*Ect*^ cells, treated with both primary and secondary antibody, demonstrated a shift in fluorescence signal; no corresponding shift signal was observed for MC58Δ*cbbA* confirming the absence of FBA on the surface of these cells. MC58Δ*cbbA cbbA* ^*EctD83A*^ cells showed clear evidence of surface‐localized FBA confirming that aldolase activity is not required for surface localization (Fig. 2).

**Figure 3:**
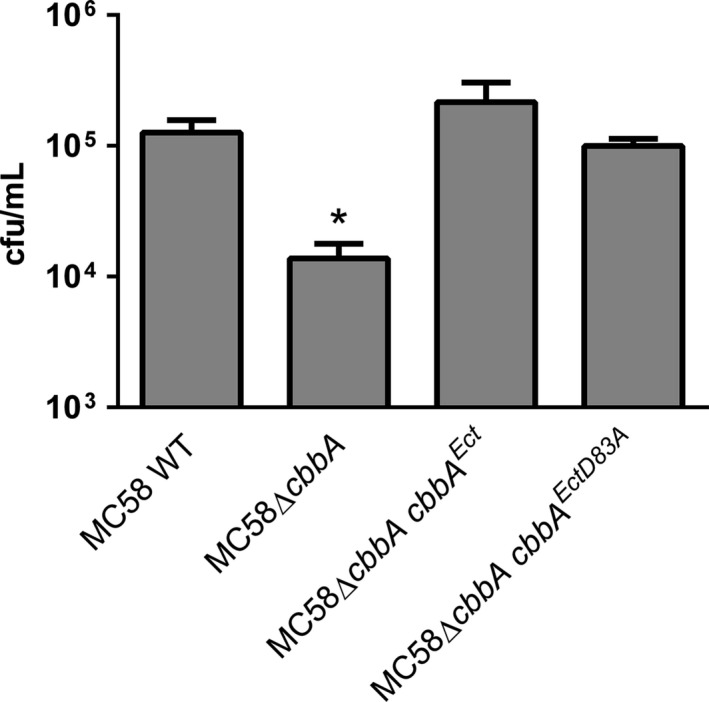
Complementation with either wild‐type *cbbA* or *cbbA* ^D83A^ restores the ability of MC58Δ*cbbA* cells to associate with human brain microvascular endothelial cells. Values shown are means from five independent experiments carried out in triplicate wells. Error bars indicate SE. * denotes *P* <0.05 (Student's *t*‐test).

The ability of these same meningococcal strains to associate with human brain microvascular endothelial cells was then examined. These experiments showed no statistically significant differences between MC58Δ*cbbA cbbA* ^*Ect*^ or MC58Δ*cbbA cbbA* ^*EctD83A*^ compared to wild‐type (Fig. 3). In contrast, MC58Δ*cbbA* showed significantly reduced association with HBME cells in line with previous results (Tunio et al. 2010b). Taken together, our data suggest that the aldolase activity of meningococcal FBA is not required for outer membrane targeting, surface localization or anchoring of FBA on the meningococcal surface, nor is it required for optimal association of *N. meningitidis* with human endothelial cells.

### Terminal lysine‐dependent plasminogen binding by meningococcal FBA

**Figure 4:**
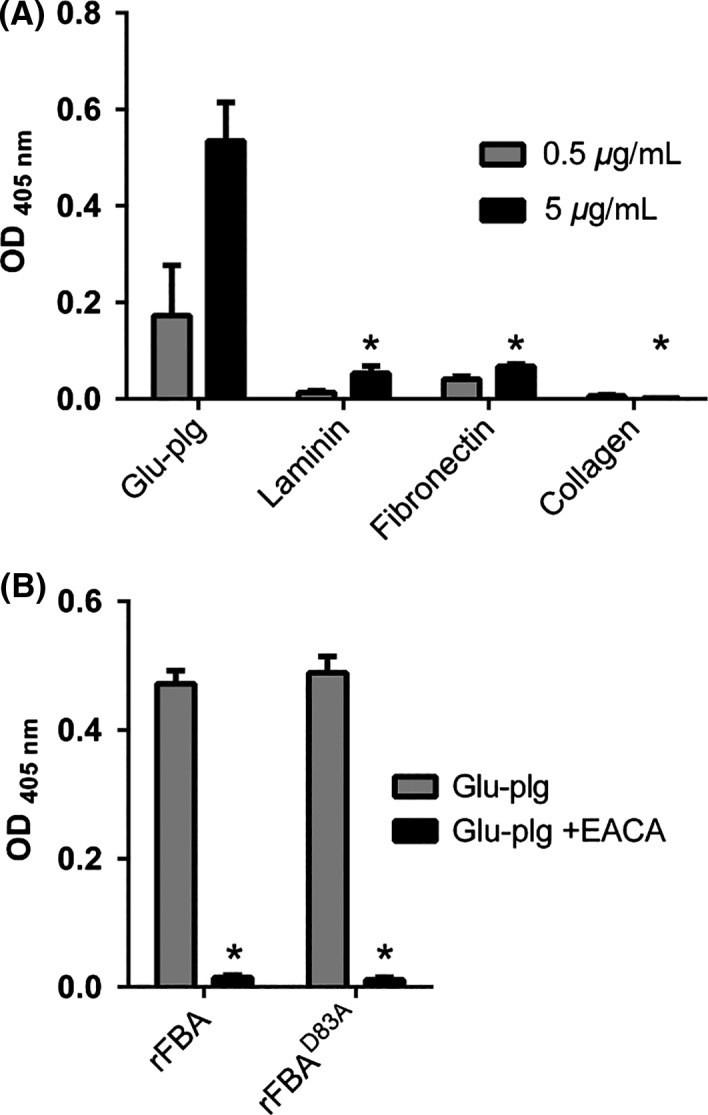
Meningococcal Fructose‐1,6‐bisphosphate aldolase (FBA) is a plasminogen‐binding protein. (A). rFBA exhibits significantly higher levels of binding to immobilized glu‐plg than to laminin, fibronectin, or collagen. Means are from three independent experiments carried out in triplicate wells. rFBA used at 5 *μ*g mL^−1^; solid‐phase ligands at 0.5 or 5 *μ*g mL^−1^. Error bars indicate SE. * denotes *P* value <0.05 (Student's *t*‐test) compared to binding of rFBA to glu‐plg (5 *μ*g mL^−1^). (B). Binding of glu‐plg to immobilized rFBA is inhibited by ε‐aminocaproic acid (5  mmol L^−1^; EACA) suggesting the involvement of FBA lysine residues in the interaction. FBA^D83A^ retains glu‐plg binding activity, confirming that aldolase activity is not required for binding. Means are from three independent experiments carried out in triplicate wells. Error bars indicate SE. * denotes *P* value <0.05 (Student's *t*‐test) compared to binding in the absence of EACA.

**Figure 5:**
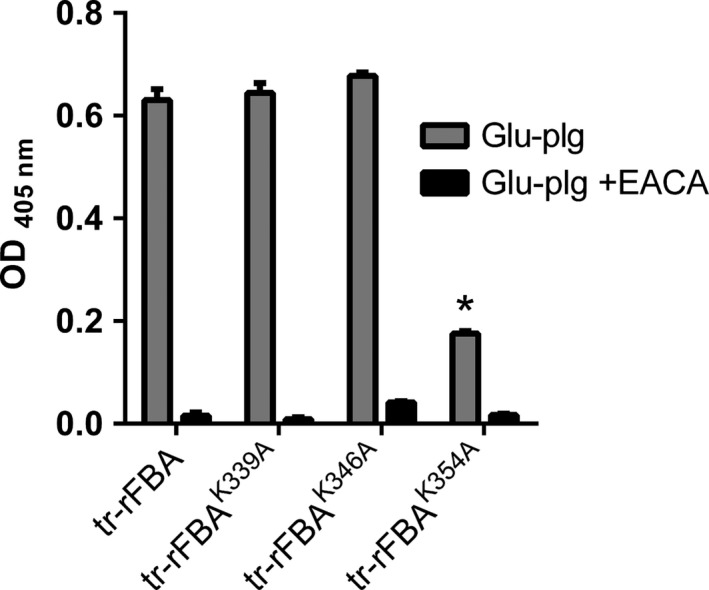
Fructose‐1,6‐bisphosphate aldolase (FBA) interacts with plasminogen via its terminal lysine residue (^354^K). Replacement of FBA residue ^354^K (tr‐rFBA^K354A^), but not residues ^339^K (tr‐rFBA^K339A^) or ^346^K (tr‐rFBA^K346A^), leads to a significantly reduced ability to bind glu‐plg. Values shown are means from three independent experiments carried out in triplicate wells. Error bars indicate SE. * denotes *P* value <0.05 (Student's *t*‐test) compared to binding of glu‐plg to tr‐rFBA in the absence of EACA.

Bacterial moonlighting proteins have been shown to bind a variety of host proteins (Henderson et al., 2011). We screened the ability of rFBA to bind to glu‐plg, fibronectin, laminin, and collagen; rFBA bound significantly to each of these substrates in a dose‐dependent manner but binding to glu‐plg occurred at much higher levels than to the other proteins (Fig. 4A). Binding to glu‐plg could be inhibited by the lysine analog, ε‐aminocaproic acid (EACA), suggesting the involvement of one or more lysine residues (Fig. 4B); no significant difference in glu‐plg binding was apparent in assays utilizing rFBA^D83A^ confirming that aldolase activity was not required for the interaction with glu‐plg (Fig. 4B). Glu‐plg was also shown to bind to a truncated derivative of rFBA containing the C‐terminal 123 amino acids of rFBA (tr‐rFBA; Fig. 5). Given the likely involvement of lysine residues in the interaction, subterminal (^339^K and ^346^K), and terminal (^354^K) lysine residues were replaced with alanine in tr‐rFBA using site‐directed mutagenesis and the effects on glu‐plg binding examined. Only the tr‐rFBA^K354A^ derivative exhibited a significantly reduced ability to bind glu‐plg confirming a critical role for the terminal lysine residue of meningococcal FBA in plasminogen binding (Fig. 5).

### Plasminogen binds to the surface of *N. gonorrhoeae* and commensal neisseriae

**Figure 6:**
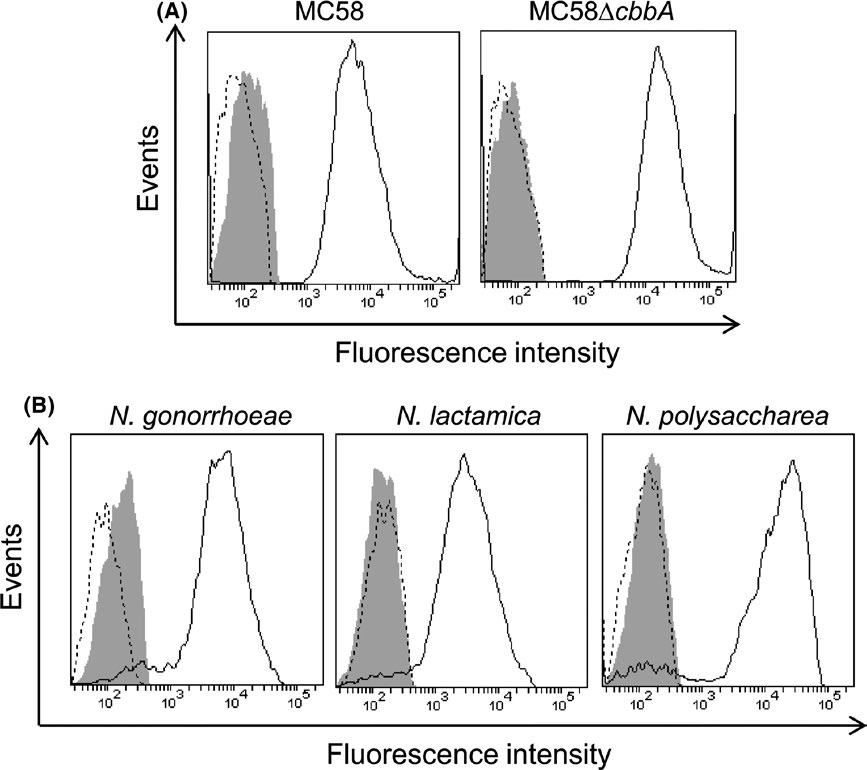
Flow cytometric analysis demonstrating the binding of plasminogen to the surface of neisserial cells. Binding of glu‐plg was detected by goat anti‐plasminogen antibody and donkey anti‐goat IgG‐Alexa Fluor488. Fluorescence is displayed as a histogram: area enclosed by the dashed line indicates bacteria treated with primary antibody only; area shaded in gray indicates bacteria treated with secondary only; area enclosed by the solid line indicates bacteria treated with both antibodies.(A). Glu‐plg binds equally to the surface of wild‐type MC58 and MC58Δ*cbbA* cells. (B). Glu‐plg binds to the surface of *N. gonorrhoeae* FA1090, *N. lactamica* ATCC 23970 and a clinical isolate of *N. polysaccharea*.

Glu‐plg was shown to bind equally to the surface of wild‐type MC58 and MC58Δ*cbbA* cells (94.6% for MC58; 92.5% for MC58Δ*cbbA*, respectively), suggesting considerable redundancy in plg binding to the bacterial surface (Fig. 6A). Glu‐plg was also shown to bind to the surface of *N. gonorrhoeae* FA1090, *N. lactamica* ATCC 23970 and a clinical isolate of *N. polysaccharea* (Fig. 6B; all ≥90%).), suggesting that the cell surface binding of plg is a generalized phenomenon among neisserial species.

## Discussion

We previously reported that FBA, a highly conserved class IIB aldolase, is localized to the outer membrane of *N. meningitidis* and is surface accessible to anti‐FBA antibodies (Tunio et al. 2010b). Furthermore, an FBA‐deficient mutant showed reduced adherence to human epithelial and endothelial cells. One question that arises from these observations is whether the enzymatic activity of the protein is required for any aspect of the moonlighting process. More specifically, is aldolase activity a prerequisite for surface localization, surface anchoring or the function(s) of FBA on the bacterial surface? In an attempt to address these questions, recombinant wild‐type and mutant derivatives of rFBA were expressed and purified under nondenaturing conditions and their respective aldolase activity determined. Mutation of the conserved aspartic acid residue in the predicted cation‐binding site abolished the aldolase activity of rFBA. This result is in agreement with similar studies on the aldolase of *E. coli* which showed that the conserved aspartic acid residue is required for polarization of the carbonyl group of glyceraldehyde 3‐phosphate during catalysis (Plater et al. 1999). We introduced ectopic copies of either an intact *cbbA* gene or a derivative harboring the mutation identified above into a *cbbA* null mutant strain of *N. meningitidis*. Proteins expressed from either gene were detected on the meningococcal surface indicating that aldolase activity is not required for transportation of FBA to the surface of meningococcal cells or its stable anchoring on the cell surface.

Fructose‐1,6‐bisphosphate aldolase expression is highly conserved in the family neisseriae; the protein being expressed by both pathogenic and nonpathogenic species (Tunio et al. 2010b). However, it remained to be determined whether FBA was localized to the surface in different strains of the meningococcus or other species of *Neisseria*. Our data suggest that the surface localization of FBA is likely to be ubiquitous among neisseriae. Interestingly, reduced fluorescence intensity was seen for two MenC strains compared to MenB, MenX, and MenZ strains. Analysis of additional strains will be required to determine whether this is a generalized phenomenon for MenC strains due to the nature of the polysaccharide capsule or results from differences in the level of capsular expression in these strains. Significantly, the presence of FBA on the surface of nonpathogenic commensal species of neisseriae suggests that the function of surface FBA might be of particular importance for the colonization of host epithelial cells. In *Streptococcus pneumoniae*, FBA functions as an epithelial cell adhesion and the host cell ligand was putatively identified as Flamingo cadherin receptor (Blau et al. 2007). Whether meningococcal FBA can also interact with this receptor will require further work.

Fructose‐1,6‐bisphosphate aldolase has also been found on the surface of *M*. *tuberculosis*, where it binds plg and, after conversion of plg to plasmin, protects the latter from the regulatory effect of *α*2–antiplasmin (de la Paz Santangelo et al. 2011). The presence of plg‐binding proteins, identified as enolase, DnaK, and peroxiredoxin on the surface of *N. meningitidis* has previously been reported (Knaust et al. 2007). Knaust et al. also noted the presence of additional, but unidentified, plg‐binding proteins in the meningococcus. Although FBA was not identified in that study, one of the unidentified plg‐binding proteins had an apparent molecular weight of *ca*. 35‐kDa, consistent with the predicted molecular weight of 38 kDa for FBA (Knaust et al. 2007). Based on this report, and the fact that FBAs from a diverse range of pathogens including *Candida albicans* (Crowe et al. 2003), *Cryptococcus neoformans* (Stie et al. 2009), *M. tuberculosis* (de la Paz Santangelo et al. 2011) and most recently *Paracoccidioides* (Chaves et al. 2015) have previously been shown to bind plg, we hypothesized that meningococcal FBA would also bind human plg. Our data confirm that meningococcal FBA binds plg in a dose‐dependent manner, but only very low levels of binding to fibronectin, laminin, or collagen were observed. Further work would be required to determine whether meningococcal FBA binds to additional host molecules.

We examined the binding of plg to wild‐type *N. meningitidis* MC58 and the *cbbA* null mutant. Wild‐type and *cbbA* null mutant cells were able to bind plg at a similar level, indicating that, as expected, there is significant redundancy in surface binding of plg. Knaust et al. similarly reported that deletion of peroxiredoxin did not have a detectable effect on surface plg binding. In the same study, despite several attempts, enolase or DnaK null mutant strains could not be generated, suggesting that these proteins are essential for cell viability, in addition to their moonlighting role on the cell surface. Furthermore, due to the high concentration of plg in human serum, all plg receptors on meningococcal cells are likely to be saturated. Consequently, the number of surface‐exposed receptors, and their available binding epitope(s), is important for this interaction, while the binding affinity is likely to be less important (Knaust et al. 2007).

As previously reported (Ullberg et al. 1992), we confirmed that *N. gonorrhoeae* can also bind plg, and for the first time demonstrated plg binding by the commensal species, *N. lactamica,* and *N. polysaccharea*. The occurrence of anchorless glycolytic enzymes on the surface of pathogenic bacterial species and their interaction with plg and other human proteins is well established, but only recently has it become evident that similar strategies are adopted by commensal species. For example, enolase was reported as a surface receptor for plg in four *Bifidobacterium* spp., (Candela et al. 2009) and *Lactobacillus plantarum* enolase binds both fibronectin and plg (Vastano et al. 2013). Binding of plg may aid the survival of neisseriae on epithelial surfaces and potentially enhance the virulence of *N. meningitidis*. Once plg is activated to plasmin, cells gain an enhanced capability to degrade fibrin clots, extracellular matrix proteins or components of the innate immune system (Lähteenmäki et al. 2001). However, Sijbrandi et al. found that Pbp, a protein exposed on the surface of *Bacteroides fragilis*, can bind plg which is not then converted to plasmin, suggesting that sequestering plg on the bacterial surface may have other advantages (Sijbrandi et al. 2008). Similarly, triosephosphate isomerase (TPI) on the cell surface of *Staphylococcus aureus* decreases activation of plg bound by enolase (Furuya and Ikeda 2011). Future work will examine whether plg bound by FBA is activated to plasmin or is retained on the bacterial surface in the inactive precursor form.

Having shown that meningococcal FBA is able to bind plg, we investigated which amino acid residues in FBA were required for optimal interaction. The binding we observed was sensitive to the lysine analog EACA, suggesting that certain lysine residues in FBA were responsible for the interaction. Knaust et al. reported that internal lysine residues (rather than a terminal lysine) were important for plg binding by enolase, DnaK, and peroxiredoxin (Knaust et al. 2007). Conversely, our site‐directed mutagenesis approach demonstrated that the terminal lysine of FBA was primarily responsible for plg binding. Importantly, replacement of the terminal lysine with alanine did not completely abolish binding and the residual binding was still abolished in the presence of EACA, suggesting that other lysine residues within FBA may contribute to the interaction with plg. Mutation in the cation‐binding site did not affect binding confirming that aldolase activity is not required for the plg‐binding activity.

The neisseriae are, to date, the only Gram‐negative bacteria in which surface‐localized FBA has been described. However, FBA orthologs are present in other pathogenic Gram‐negative bacterial species including, for example, *Pseudomonas aeruginosa* and *Acinetobacter baumanii*. Interestingly, Flores‐Ramirez et al. recently reported the presence of FBA in the cell wall fraction of *Coxiella burnetii*, suggesting the possibility that translocation of FBA to the Gram‐negative cell envelope might be a more generalized phenomenon (Flores‐Ramirez et al. 2014). Finally, the presence of class II FBAs in many pathogens (and the absence of a structurally or antigenically similar protein in humans) makes FBA a potential target for antimicrobial therapy (Daher et al. 2010) and a possible vaccine candidate.

## Conflict of Interest

None declared.

## Supporting information


**Table S1.** Bacterial strains used in this study.
**Table S2.** Plasmids used in this study.
**Table S3.** Primers used in this study.
