# Supplementary material for: Fructose‐1,6‐bisphosphate aldolase of Neisseria meningitidis binds human plasminogen via its C‐terminal lysine residue
Source: Microbiologyopen. 2016 Jan 5;5(2):340–50. doi: 10.1002/mbo3.331 (PMC4831477; doi:10.1002/mbo3.331)
Supplement: Supplementary file 1 — Table S1. Bacterial strains used in this study. Table S2. Plasmids used in this study. Table S3. Primers used in this study. [file MBO3-5-340-s001.docx]

Supplementary Tables

Table S1. Bacterial strains used in this study.

| Bacterial strain | Characteristics | Source or reference |
| --- | --- | --- |
| *E. coli* |  |  |
| JM109 | expression strain | Promega |
| XL10-Gold | cloning strain | Agilent Technologies |
|  |  |  |
| *N. meningitidis* |  |  |
| MC58 | B: P1.7,16-2: F1-5: ST-74 (cc32) | ([Tettelin *et al.*, 2000](#_ENREF_5)) |
| Z4667 | B: P1.18-2,1-1,1-2: F3-9: ST-48 (cc41/44) | ([Maiden *et al.*, 1998](#_ENREF_1)) |
| Z4673 | B: P1.7-2,4: F1-5: ST-41 (cc41/44) | ([Maiden *et al.*, 1998](#_ENREF_1)) |
| Z4181 | C: P1.5,2-1: F5-4: ST-11 (cc11) | ([Maiden *et al.*, 1998](#_ENREF_1)) |
| 8013 | C: P1.21,26-2: F1-5: ST-177 (cc18) | ([Rusniok *et al.*, 2009](#_ENREF_4)) |
| Z6429 | X: P1.18,25-1: F5-5: ST-39 (cc198) | ([Maiden *et al.*, 1998](#_ENREF_18)) |
| Z6428 | Z: P1.5-2,10-9: F1-5: ST-31 (cc334) | ([Maiden *et al.*, 1998](#_ENREF_18)) |
| MC58Δ*cbbA* | *cbbA* replaced with kanamycin cassette | ([Tunio *et al.*, 2010](#_ENREF_6)) |
| Z4181Δ*cbbA* | *cbbA* replaced with kanamycin cassette | This study |
| Z4667Δ*cbbA* | *cbbA* replaced with kanamycin cassette | This study |
| Z4673Δ*cbbA* | *cbbA* replaced with kanamycin cassette | This study |
| 8013Δ*cbbA* | *cbbA* replaced with kanamycin cassette | This study |
| MC58Δ*cbbA cbbA^Ect^* | MC58Δ*cbbA* containing *cbbA* and erythromycin cassette at an ectopic site | ([Tunio *et al.*, 2010](#_ENREF_6)) |
| MC58Δ*cbbA cbbA^EctD83A^* | MC58Δ*cbbA* containing *cbbA* (encoding D83A mutation) and erythromycin cassette | This study |
|  |  |  |
| Other *Neisseria* species |  |  |
| *N. gonorrhoeae* | ATCC 700825 (FA1090) | NCTC |
| *N. lactamica* | ATCC 23970 | ([Minogue *et al.*, 2014](#_ENREF_2)) |
| *N. polysaccharea* | Clinical isolate | ([Oldfield *et al.*, 2007](#_ENREF_3)) |

Table S2. Plasmids used in this study.

| Plasmid | Characteristics | Source |
| --- | --- | --- |
| pSAT-4 | Mutagenesis plasmid containing a kanamycin resistance cassette in the same orientation as the deleted *cbbA* gene | ([Tunio *et al.*, 2010](#_ENREF_6)) |
| pSAT-9 | MC58 *cbbA* gene cloned in pQE70 (C-terminal His-tag) | ([Tunio *et al.*, 2010](#_ENREF_6)) |
| pFS-2 | pSAT-9 with a single nucleotide mutation (A to C) at position 248 of *cbbA* to encode rFBA^D83A^ | This study |
| pSAT-12 | Ectopic complementation vector containing *cbbA* and an erythromycin resistance cassette flanked by two meningococcal genes (NMB0102 and NMB0103) | ([Tunio *et al.*, 2010](#_ENREF_6)) |
| pFS-5 | pSAT-12 with a single nucleotide mutation (A to C) at position 248 of *cbbA* to encode FBA^D83A^ | This study |
| pQE30 | Expression vector (N-terminal His-tag) | Qiagen |
| pFS-7 | Truncated *cbbA* gene from MC58 encoding the C-terminal fragment (^232^H to ^354^K) of FBA cloned in pQE30 | This study |
| pFS-71 | pFS-7 with nucleotide mutations (AA to GC) at positions 1015 and 1016 of *cbbA* to encode tr-rFBA^K339A^ | This study |
| pFS-72 | pFS-7 with nucleotide mutations (AA to GC) at positions 1036 and 1037 of *cbbA* to encode tr-rFBA^K346A^ | This study |
| pFS-73 | pFS-7 with nucleotide mutations (AA to GC) at positions 1060 and 1061 of *cbbA* to encode tr-rFBA^K354A^ | This study |

Table S3. Primers used in this study.

| Primer | Sequence^a^ | Restriction site |
| --- | --- | --- |
| FBA_D83AF | CGTCATGCACCAAGCCACGGCGCATCAC |  |
| FBA_D83AR | GTGATGCGCCGTGGGCTTGGTGCATGACG |  |
| CterFBAF1 | CGC**GGATCC**CACGGCTCCAGCTCCGTTCCG | BamHI |
| CterFBAR1 | CGC**GGATCC**TTATTTGACGATTTGGTTCAATTCG | BamHI |
| FBA_K339F | CAAACCTGTTTCGTTGGAAGCAATGGCAAGCCGTTATGCC |  |
| FBA_K339R | GGCATAACGGCTTGCCATTGCTTCCAACGAAACAGGTTTG |  |
| FBA_K346F | AATGGCAAGCCGTTATGCCGCGGGCGAATTGAACCAAATC |  |
| FBA_K346R | GATTTGGTTCAATTCGCCCGCGGCATAACGGCTTGCCATT |  |
| FBA_K354F | CGAATTGAACCAAATCGTCGCATAAGGATCCGCATGCGAG |  |
| FBA_K354R | CTCGCATGCGGATCCTTATGCGACGATTTGGTTCAATTCG |  |

^a^ All primers were designed from the *N. meningitidis* MC58 genome sequence. Sequences in bold identify restriction enzyme sites.

References

Maiden, M.C., Bygraves, J.A., Feil, E., Morelli, G., Russell, J.E., Urwin, R.*, et al.* (1998) Multilocus sequence typing: a portable approach to the identification of clones within populations of pathogenic microorganisms. *Proc Natl Acad Sci U S A* **95:** 3140-3145.

Minogue, T.D., Daligault, H.A., Davenport, K.W., Bishop-Lilly, K.A., Bruce, D.C., Chain, P.S.*, et al.* (2014) Draft genome assembly of *Neisseria lactamica* type strain A7515. *Genome Announc* **2:** e00951-00914.

Oldfield, N.J., Bland, S.J., Taraktsoglou, M., Ramos, F.J.D., Robinson, K., Wooldridge, K.G., and Ala'Aldeen, D.A.A. (2007) T-cell stimulating protein A (TspA) of *Neisseria meningitidis* is required for optimal adhesion to human cells. *Cell Microbiol* **9:** 463-478.

Rusniok, C., Vallenet, D., Floquet, S., Ewles, H., Mouze-Soulama, C., Brown, D.*, et al.* (2009) NeMeSys: a biological resource for narrowing the gap between sequence and function in the human pathogen *Neisseria meningitidis*. *Genome Biol* **10:** R110.

Tettelin, H., Saunders, N.J., Heidelberg, J., Jeffries, A.C., Nelson, K.E., Eisen, J.A.*, et al.* (2000) Complete genome sequence of *Neisseria meningitidis* serogroup B strain MC58. *Science* **287:** 1809-1815.

Tunio, S.A., Oldfield, N.J., Berry, A., Ala'Aldeen, D.A., Wooldridge, K.G., and Turner, D.P. (2010) The moonlighting protein fructose-1, 6-bisphosphate aldolase of *Neisseria meningitidis*: surface localization and role in host cell adhesion. *Mol Microbiol* **76:** 605-615.
